# The Sleep Inbred Panel, a Collection of Inbred *Drosophila melanogaster* with Extreme Long and Short Sleep Duration

**DOI:** 10.1534/g3.118.200503

**Published:** 2018-07-10

**Authors:** Yazmin L. Serrano Negron, Nancy F. Hansen, Susan T. Harbison

**Affiliations:** * Laboratory of Systems Genetics, National Heart Lung and Blood Institute, National Institutes of Health, Bethesda, MD; † Comparative Genomics Analysis Unit, National Human Genome Research Institute, National Institutes of Health, Rockville, MD

**Keywords:** sleep, *Drosophila melanogaster*, whole-genome sequence

## Abstract

Understanding how genomic variation causes differences in observable phenotypes remains a major challenge in biology. It is difficult to trace the sequence of events originating from genomic variants to changes in transcriptional responses or protein modifications. Ideally, one would conduct experiments with individuals that are at either extreme of the trait of interest, but such resources are often not available. Further, advances in genome editing will enable testing of candidate polymorphisms individually and in combination. Here we have created a resource for the study of sleep with 39 inbred lines of *Drosophila*—the Sleep Inbred Panel (SIP). SIP lines have stable long- and short-sleeping phenotypes developed from naturally occurring polymorphisms. These lines are fully sequenced, enabling more accurate targeting for genome editing and transgenic constructs. This panel facilitates the study of intermediate transcriptional and proteomic correlates of sleep, and supports genome editing studies to verify polymorphisms associated with sleep duration.

Genomic studies of wild-derived populations of flies have identified thousands of polymorphisms that affect morphological, physiological, and behavioral complex traits (Ayroles *et al.* 2009; Jordan *et al.* 2012; Mackay *et al.* 2012; Weber *et al.* 2012; Chow *et al.* 2013; Harbison *et al.* 2013; Ellis *et al.* 2014; Vaisnav *et al.* 2014; Arya *et al.* 2015; Ayroles *et al.* 2015; Dembeck *et al.* 2015a; Dembeck *et al.* 2015b; Gaertner *et al.* 2015; Garlapow *et al.* 2015; Ivanov *et al.* 2015; Montgomery *et al.* 2015; Morgante *et al.* 2015; Morozova *et al.* 2015; Shorter *et al.* 2015; Unckless *et al.* 2015; Zwarts *et al.* 2015; Chow *et al.* 2016; He *et al.* 2016; Hunter *et al.* 2016; Vonesch *et al.* 2016; Harbison *et al.* 2017; Lobell *et al.* 2017; Wu *et al.* 2018). A challenging next step is to demonstrate how the polymorphisms associated with a trait influence phenotype (Mackay *et al.* 2009; Albert and Kruglyak 2015). One potential approach is to measure the phenotypic, transcriptional, and proteomic impact of perturbing candidate polymorphisms, a strategy that has become possible with the advent of genome editing (Bassett and Liu 2014; Albert and Kruglyak 2015). Such perturbations are best made in consistent genetic backgrounds, where one can accurately estimate enhancing and suppressing epistatic effects (Yamamoto *et al.* 2008; Swarup *et al.* 2012; Mackay 2014). Here we developed a 39-line panel of inbred flies having extreme long and short sleep duration, which we refer to as the Sleep Inbred Panel (SIP). Because the SIP lines have extreme differences in phenotype, advanced intercross population designs developed from two or more strains could be employed to identify context-dependent pleiotropic loci or genetic modifiers (Lawson *et al.* 2011; Huang *et al.* 2012; Kislukhin *et al.* 2013; Swarup *et al.* 2013; King *et al.* 2014; Najarro *et al.* 2015; Shorter *et al.* 2015; Chow *et al.* 2016; Chandler *et al.* 2017). The SIP is therefore a useful tool for the design of genome modifications, the identification of phenotypic, transcriptional, and proteomic correlates, and the understanding of context-dependent effects.

## Materials and Methods

### Construction of the Sleep Inbred Panel

**Figure 1:**
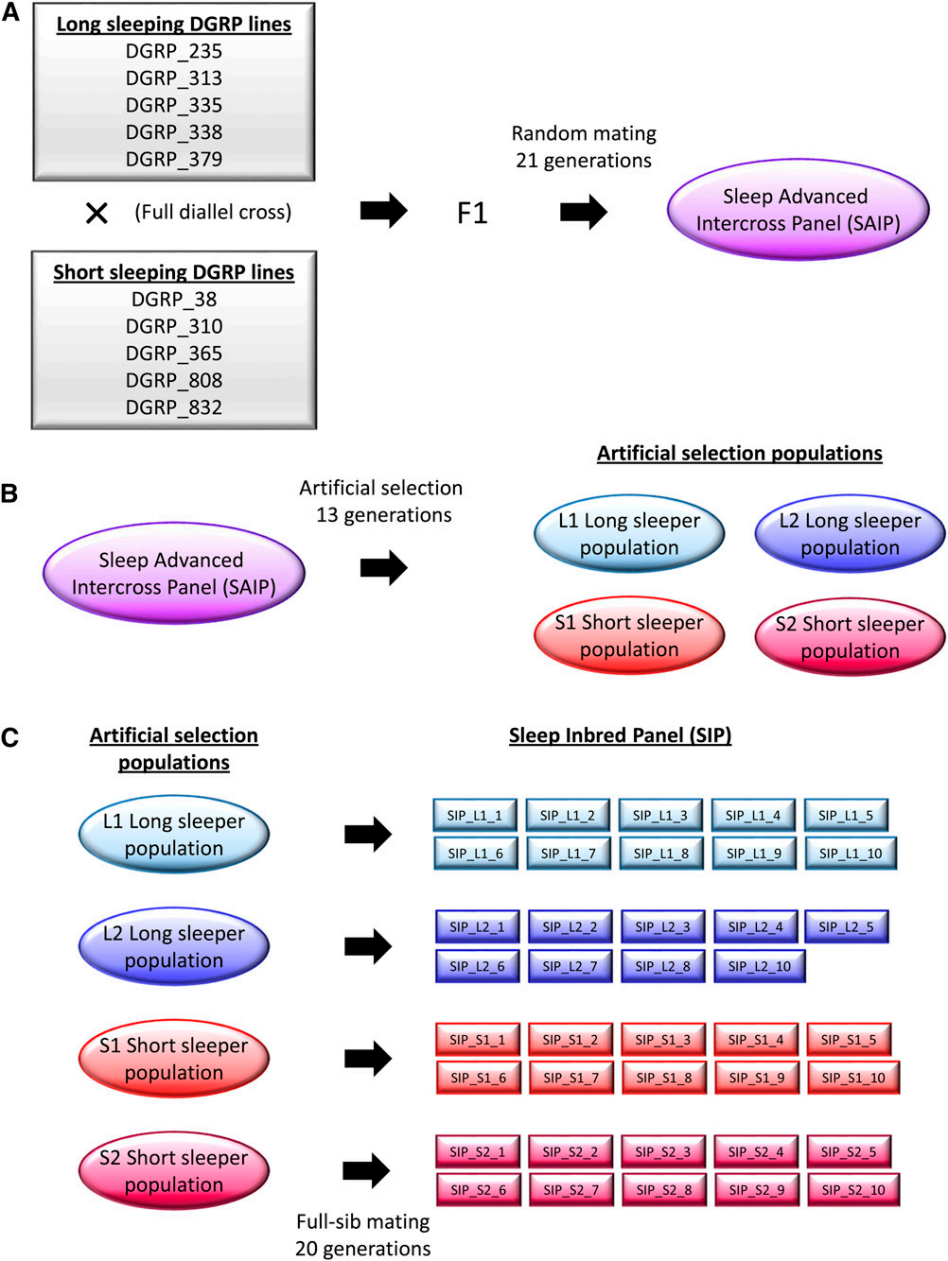
Construction of the Sleep Inbred Panel. The steps used to construct the SIP are indicated. (A) Step 1 is the construction of the Sleep Advanced Intercross Population (SAIP) using 10 long- and short-sleeping inbred lines. The F1 of a full diallel cross was randomly mated for 21 generations to produce the outbred SAIP. (B) Step 2 is an artificial selection protocol applied to the SAIP to produce two replicate long- and two replicate short-sleeping populations of flies. (C) Step 3 is the construction of the SIP via 20 generations of full-sib mating.

The process for construction of the Sleep Inbred Panel is outlined in Figure 1 and involves three major steps. The first two steps were done previously (Harbison *et al.* 2017), but we outline them here briefly. The first step was the construction of an outbred population of flies, the Sleep Advanced Intercross Population (SAIP) using ten lines from the *Drosophila* Genetic Reference Panel (DGRP) (Mackay *et al.* 2012; Huang *et al.* 2014) with the most extreme night sleep phenotypes in both sexes (Figure 1A). The five lines with shortest average night sleep were DGRP_38, DGRP_310, DGRP_365, DGRP_808, and DGRP_832 (Harbison *et al.* 2013). The five lines with the longest average night sleep were DGRP_235, DGRP_313, DGRP_335, DGRP_338, and DGRP_379 (Harbison *et al.* 2013). All ten lines were crossed in a full diallel design, resulting in 100 crosses. We randomly assigned two virgin females and two males from the F1 of each cross into 20 bottles, with 10 males and 10 females in each bottle. At each subsequent generation, we randomly mixed 20 virgin females and 20 males from each bottle to propagate the next generation. Each generation of random mating had a census population size of 800. We continued this random mating scheme for 21 generations, resulting in the SAIP (Harbison *et al.* 2017).

The second step was to select for long and short night sleep duration (Figure 1B). To do this we split the SAIP into four populations by seeding four bottles with 25 randomly chosen flies of each sex. Two populations were selected for long night sleep (L1 and L2), and two populations were selected for short night sleep (S1 and S2) using the following artificial selection procedure each generation. We measured sleep and activity over a 5-day period in 100 virgins of each sex from each population. The 25 males and females with the highest (lowest) night sleep within each population were chosen as parents for the next generation of long (short) sleepers. We repeated this procedure for 13 generations. This resulted in two short-sleeper populations with average night sleep of 111.9 ± 10.74 min. and 54.8 ± 5.66 min. for replicate 1 and replicate 2, respectively, and two long-sleeper populations with average night sleep of 685.0 ± 3.35 min. and 678.5 ± 3.46 min. for replicate 1 and 2, respectively (Harbison *et al.* 2017). After Generation 13, the flies were maintained for each population/replicate via random mating for 17 generations.

The third step was the creation of the Sleep Inbred Panel (Figure 1C). At generation 51, the long- and short-sleeping selected populations were used to create inbred lines. We created 10 lines each from the L1, S1, and S2 populations, and 9 lines from the L2 population (39 lines total). Each line was created using a single male and a single female from one of the populations to start the line; one male and one female from the progeny were used to propagate the line to the next generation. Full-sib mating continued in this manner for 20 generations. Inbred stocks were maintained past generation 20 by random mating.

### Rearing and assay conditions

For culturing and sleep assays, flies were reared in a single incubator under standard conditions (25°, 60% humidity, 12:12 hr light:dark cycle) on standard *Drosophila* medium (https://bdsc.indiana.edu/information/recipes/bloomfood.html). Prior to sleep assays, male and female flies were collected as virgins and aged to 4 – 7 days in same-sex vials of 20 flies each to standardize mating status and social exposure (Ganguly-Fitzgerald *et al.* 2006; Isaac *et al.* 2010). For sleep assays, lines were randomly assigned to one of four blocks: three blocks had ten lines, and one block had nine lines. Sleep assays were replicated twice for each SIP line. The first replicate was measured in the generation immediately following the inbreeding procedure, while the second replicate was measured two generations later. We did not observe any differences in night sleep among replicate measures (Table S1). A total of 32 flies/sex/line were measured.

### Sleep phenotyping

We measured sleep and activity in the SIP in the rearing and assay conditions stipulated above. Individual virgin males and females were placed into Drosophila Activity Monitoring System (DAM2) monitors (Trikinetics, Waltham, MA) under CO_2_ anesthesia. Activity counts were recorded for the subsequent seven days; the first day of data were discarded as the flies were recovering from CO_2_ and acclimating to the monitor tubes. At the end of the seven-day period, each fly was visually examined; data from flies that did not survive the duration of the monitoring period was discarded. We used a C# program (R. Sean Barnes) to calculate the sleep duration, the number of sleep bouts, and the average sleep bout length during the day or night; the waking activity, which is the number of activity counts divided by the number of minutes spent awake in a 24-hour period; and the sleep latency, which is the amount of time before the first night sleep bout.

### Phenotypic data analysis

Lines of the SIP originate from four different selection populations: L1 and L2, which were two replicate populations selected for long sleep; and S1 and S2, the two replicate populations selected for short sleep. We first analyzed the sleep parameters for their differences among selection scheme and replicate population within selection scheme using the ANOVA model *Y* = *µ* + *Sel* + *Reppop*(*Sel*) + *Sex* + *Rep* + *Sex*×*Reppop*(*Sel*) + *Rep*×*Reppop*(*Sel*) + *Sex*×*Reppop*×*Rep*(*Sel*) + *ε*, where *Sel* is selection scheme, *Reppop* is replicate population, *Rep* is phenotypic replicate, and *ε* is the error term. There were significant differences in sleep phenotypes among selection schemes and replicate populations. Next, we compared the mean sleep of each SIP line with the mean of its progenitor population (*i.e.*, the artificially selected population from which each SIP line was derived) using the ANOVA model *Y* = *µ* + *Sex* + *Line* + *Rep* + *Sex*×*Line* + *Sex*×*Rep* + *Line*×*Rep* + *Line*×*Sex*×*Rep* + *ε*, where *Rep* and *ε* are as defined above. We used post-hoc Tukey comparisons to determine which lines were significantly different from the progenitor.

### DNA extraction and sequencing

Two replicates of thirty female flies were flash-frozen from each line. DNA was extracted using a cell lysis solution [1.58 g of Tris-HCl (Quality Biological, Gaithersburg, MD), 37.22 g EDTA disodium salt (Quality Biological, Gaithersburg, MD) and filled to 1 liter with RNase/DNase-free water, adjusting the pH to 8.0 with 10 M NaOH (Sigma Aldrich, St. Louis, MO) when necessary]. Flies were homogenized using an Omni Bead Ruptor (Omni International, Kennesaw, GA). The solution was incubated with 10% SDS (Thermo Fisher Scientific, Waltham, MA) and 20 mg/mL Proteinase K (Thermo Fisher Scientific, Waltham, MA) at 65° for 1 hr. The lysate was RNase A treated (20 mg/ml) (Thermo Fisher Scientific, Waltham, MA) by mixing and incubating at 37° for 15 min. Ammonium Acetate (Quality Biological, Gaithersburg, MD) solution was added to samples chilled on ice for 5 min to precipitate proteins. 100% isopropanol (VWR International, Radnor, PA) was added and mixed to precipitate the DNA; samples were incubated for 1 hr at -20°. The DNA pellet was washed with 75% ethanol (NIH Supply Center, Gaithersburg, MD), then re-hydrated in RNase/DNase-free water. DNA samples were then purified using phenol-chloroform extraction. We diluted each DNA sample with 10mM Tris (Quality Biological, Gaithersburg, MD), 1mM EDTA, pH 7.8 to bring sample volume to 200 µL. Next, 200 µL of phenol:chloroform:isoamyl alcohol (25:24:1) (Sigma Aldrich, St. Louis, MO) was added to each sample. We then centrifuged samples and transferred the aqueous phase to a new 1.5mL tube. We added 200uL of chloroform (NIH Supply Center, Gaithersburg, MD) to each tube, centrifuged samples and transferred the upper aqueous layer to a new 1.5mL tube. Next, DNA precipitation was initiated by adding 20 μL of sodium acetate (NaOAc) (Sigma Aldrich, St. Louis, MO), 500 μL of ethanol, and 1 μL of glycogen. Samples were then placed on ice, centrifuged at maximum speed for 30 min, and then the supernatant was discarded. We washed the pellet with 500 uL of ethanol and centrifuged samples for 5 min. Afterward, we removed the supernatant and dissolved the pellet in 25 μL sterile 10mM Tris, 0.1mM EDTA, pH 7.8. The samples were heated for 2 min at 55°. We measured DNA concentration and quality with Nanodrop 8000 (Thermo Fisher Scientific, Waltham, MA).

### Tru-Seq PCR-Free Library Method

For all lines save one, two micrograms of genomic DNA were sheared to ∼550 bp using a Covaris E220 with settings: duty cycle 10%; intensity 175; cycles/burst 200; and time 80s. Only one microgram of DNA was available for line SIP_L2_2, so the DNA was sheared to ∼350 bp using a Covaris E220 with settings: duty cycle 10%; intensity 3; cycles/burst 200; and time 60s. Libraries were constructed using the Tru-Seq DNA PCR-Free LT Sample Prep Kit (Illumina, San Diego, CA) according to the manufacturer’s protocol. The libraries were pooled and run on an Illumina HiSeq 2500 with version 3 sequencing reagents to generate a minimum of 10 million paired-end 251-base reads per library (Illumina, San Diego, CA), resulting in 30-50X genome coverage on average (Figure S1). The HiSeq data were processed using RTA1.18.64 and CASAVA 1.8.2.

### Sequence processing, alignment, and variant calls

All sequence reads were aligned to *D. melanogaster* assembly BDGP Release 6, UCSC version dm6 (obtained from UCSC Genome Browser FTP site). Alignments were performed using two programs: BWA-MEM version 0.7.12 (Li 2013) and Novoalign version 3.02.07 (Novocraft Technologies, Selangor, Malaysia), using the -t 400 option to optimize alignment speed. PCR duplicates were removed from all aligned read sets using samtools version 0.1.17 (Li *et al.* 2009). Read groups were added to BWA alignments, which were then realigned around known indels from the set of DGRP Freeze 2 polymorphisms (Huang *et al.* 2014) using GATK version 2.8.1 (Van der Auwera *et al.* 2013). Confirmation of sex for each sample was performed by calculating the ratio of the average read depth on the *X* chromosome to the average read depth on chromosome *2L*. The ratio of average read depth on the *X* chromosome to that of chromosome *2L* was greater than 0.96 for every line except for SIP_L2_2, which had a ratio of 0.50. Thus, SIP_L2_2 DNA likely originated from male flies (both sexes were collected for DNA). All variants were called by running LoFreq version 2.1.2 (Wilm *et al.* 2012), run with the default parameter statement “lofreq call-parallel–pp-threads 8 -f dm6.fa -o lofreq.out.vcf reads.bam”, where “dm6.fa” is the *D. melanogaster* 6.0 reference sequence file, “lofreq.out.vcf” is the output file, and “reads.bam” is the BAM file aligned reads (either BWA or Novoalign). The call-parallel feature of LoFreq was invoked to call all variants, rare or common. Allele counts for all single nucleotide variant sites were determined using the “bamcounts” command of the bardCNV package (http://github.com/nhansen/BardCNV) with the option -minqual 20 to filter reads for a minimum phred quality of 20 (Table S2). Counts of reads spanning indels were performed by first widening indel variants to their narrowest unambiguous region, then tallying reads with and without the indel using the perl module Bio::SamTools. Confidence intervals with the highest posterior density interval for the estimated read allele proportions were calculated in R using the CRAN “binom” package’s “binom.bayes” function (https://CRAN.R-project.org/doc/FAQ/R-FAQ.html). We plotted LoFreq quality score distributions for known DGRP founder alleles and novel predictions (Figure S2). Using this plot, we set a quality score threshold of 1000 for the novel predicted calls; variants less than this threshold were annotated as low scoring in the final .vcf file’s “FILTER” field. We grouped variant calls into the following categories: 1) DGRP_SNP, SNP calls that match SNPs (chromosome arm, position, and alternate allele) called as present in one of the 10 DGRP founder lines (Huang *et al.* 2014); 2) DGRP_UNGENOTYPED_SNP, DGRP SNPs that had a missing entry for at least one of the 10 DGRP founder lines (Huang *et al.* 2014); 3) DGRP_FILTERED_SNP, SNPs that were part of the original 6,149,822 variants found in the DGRP but due to low quality scores did not make the final list of 4,438,427 (Huang *et al.* 2014); 4) UNMAPPED_IN_DM3, variants that fell on the Het, *U*, *4*, *M*, and *Y* chromosomes of the *D. melanogaster* 5.0 sequence (dm3) and were not part of the 4,438,427 DGRP variants; 5) DENOVO_SNP, SNPs perfectly associated with one DGRP founder haplotype and not previously known (see the Hidden Markov Model analysis below); 6) SELECTED_DENOVO: non-DGRP SNPs that were detected only on one HMM-predicted founder haplotype, but only within one selected population (*e.g.*, L1, L2, S1, or S2); 7) PUTATIVE_FALSE_POSITIVE_SNP, variants that did not meet *de novo* SNP criteria and did not fall into any other category; and 8) SNPs removed due to a LoFreq quality score less than 1000. We annotated variant calls with SnpEff version 4.3t (Cingolani *et al.* 2012).

### Mapping of founder genotypes

To predict which of the original 10 DGRP founder haplotypes are present at any genomic locus in each of the 39 SIP lines, we utilized the Hidden Markov Model (HMM) of King *et al.* (King *et al.* 2012). Our version of the model considered all DGRP polymorphic sites that are informative in the 10 founder lines and were detected as variant by LoFreq in at least one of the 39 inbred lines. We constructed 55 states: 10 homozygous states, in which both line’s homologous chromosomes derive from the same DGRP founder, and 45 heterozygous states, in which two different DGRP founders’ haplotypes are present in the line. Initiation and emission probabilities were set as in King *et al.* and transition probabilities were calculated from an empirically-derived tabulation of recombination rates (Comeron *et al.* 2012) as reported by the program RRC, version 2.3 [Fiston-Lavier AS and Petrov DA. *Drosophila melanogaster* Recombination Rate Calculator: http://petrov.stanford.edu/cgi-bin/recombination-rates_updateR5.pl]. Recombination rates at positions between those tabulated (Comeron *et al.* 2012) were estimated by linear interpolation.

To implement the HMM, we altered the Perl script made available by King *et al.* (King *et al.* 2012) to (a) read allele counts for inbred lines and genotypes of DGRP founders from tab-delimited files rather than a mysql database; (b) allow for an arbitrary number of founder lines (10 for this study); and (c) read and correctly utilize the recombination rates reported by the program RRC to calculate transition probabilities.

### Data availability

The DNA sequences have been deposited in the Sequence Read Archive under ID code SRP126512; BioProject PRJNA421951. Supplementary tables S1-S9, figures S1-S4, and File S1 have been deposited on figshare. A text file of variant calls and confidence intervals using both BWA and Novoalign sequence alignments (Files S2 and S3, respectively) and a list of annotated variants in .vcf format have been provided (Files S4 and S5, respectively) on figshare. The scripts used to conduct the HMM analysis are on github: http://github.com/nhansen/SleepInbredPanel. Sleep Inbred Panel lines are available from the Bloomington *Drosophila* Stock Center (Flybase stock numbers FBst0076271 – FBst0076309; Bloomington *Drosophila* stock center numbers 76271-76309) (Bloomington, IN). Supplemental material available at Figshare: https://doi.org/10.25387/g3.6789968.

## Results and Discussion

### Construction of the Sleep Inbred Panel

The Sleep Inbred Panel (SIP) is the result of 21 generations of outbreeding, 13 generations of artificial selection for extreme sleep duration, 17 generations of post-selection maintenance, and 20 generations of subsequent inbreeding. In a previous study, we constructed the Sleep Advanced Intercross Population (SAIP) by crossing 10 long- and short- sleeping lines of the DGRP in a full diallel cross and then allowing the progeny to mate randomly for 21 generations (Figure 1A) (Harbison *et al.* 2017). The SAIP was used to conduct an artificial selection experiment in which two populations were selected for long night sleep (L1, L2), and two populations were selected for short night sleep (S1, S2) (Figure 1B) (Harbison *et al.* 2017). Here, we have preserved the differences in sleep duration observed in the previous study by creating inbred lines from these four artificially-selected populations (Figure 1C). Each line was seeded with a single male and virgin female from one of the four selection populations. Each generation thereafter, a single male and virgin female were used to propagate each line. This full-sib inbreeding continued for 20 generations. Thirty-nine inbred lines were created: 10 lines from the L1 long sleeper population, 9 lines from the L2 long sleeper population, 10 lines from the S1 short sleeper population, and 10 lines from the S2 short sleeper population. We refer to this collection of inbred lines as the Sleep Inbred Panel (SIP).

### SIP lines have extreme sleep duration phenotypes

**Figure 2:**
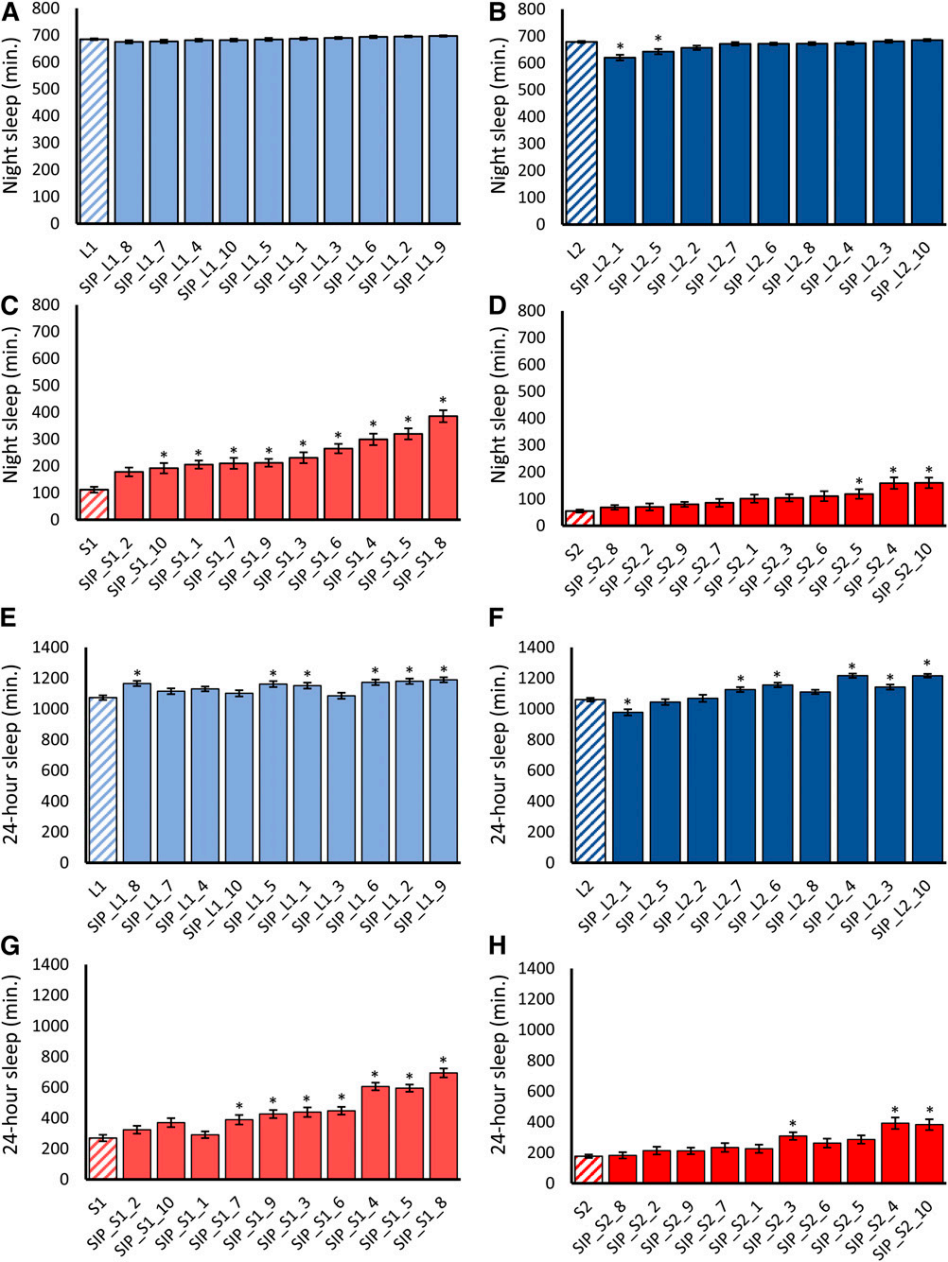
Night sleep and 24-hour sleep in the Sleep Inbred Panel. Mean night sleep ± SE is plotted for each line of the SIP as compared to the mean of its corresponding progenitor population. SIP lines are ordered from the shortest night sleep to the longest. Solid colors indicate SIP line; diagonal bars indicate the mean of the progenitor population as reported in (Harbison *et al.* 2017). (A-D) Night sleep in SIP lines derived from the (A) L1 population; (B) L2 population; (C) S1 population; and (D) S2 population. (E-H) 24-hour sleep in SIP lines derived from the (E) L1 population; (F) L2 population; (G) S1 population; and (H) S2 population. Asterisks indicate lines with sleep duration significantly different (*P* < 0.05) from the progenitor population.

Average night sleep duration in lines of the SIP ranged from 68.61 ± 8.55 min. to 697.14 ± 2.66 min. (Table S3). Differences in night sleep were evident depending upon the direction of selection in the progenitor population (*P*_selection_ = 0.0220, four-way nested ANOVA model), and varied among replicate populations selected in the same direction (*P*_replicatepopulation(selection)_ = 0.0159, four-way nested ANOVA model) (Table S1). To determine whether we had captured the extreme night sleep phenotypes present in the artificially-selected populations, we compared mean night sleep in the SIP with the mean night sleep in the progenitor artificially selected populations (Harbison *et al.* 2017). Night sleep in long-sleeper lines was equivalent to that of the L1 and L2 progenitor populations (Figure 2A and 2B; Table S4), except for two L2-derived lines that had significantly reduced sleep (Figure 2B). Night sleep was significantly increased in every line derived from the S1 short sleeper population except for SIP_S1_2 (*P*_Line_ = 0.0098, 3-way ANOVA model; Figure 2C), however. This result indicated that either inbreeding had not completely captured the short sleep in the S1 population, or that some of the extreme short sleep phenotypes were lost during the 17-generation maintenance period, possibly due to natural selection against short sleep. In contrast, night sleep in lines derived from the S2 population was equivalent to the S2 progenitor population, with the exception of three lines (*P*_Line_ = ns, 3-way ANOVA; Figure 2D). Similar results were observed if 24-hour sleep duration was considered (Figure 2E-H), though there were more differences among lines (Table S4; see Figure S3 for day sleep phenotypes). Furthermore, night, day, and 24-hour sleep were stable across three generations—that is, replicate 1 and replicate 2 of the sleep measurements (*P*_rep_ = ns for these three phenotypes) (Table S1). Thus, we largely preserved the extreme long- and short-sleeping night sleep phenotypes that we observed in the original selection populations; interestingly, significant differences from the original selection population means tended to be increases in sleep. Inbred lines derived from the S2 population had 24-hour average sleep duration that was as low if not lower than that of previously identified single-gene mutations and wild-derived inbred lines. Females of SIP_S2_1, SIP_S2_2, SIP_S2_4, SIP_S2_5, SIP_S2_6, SIP_S2_7, SIP_S2_8, and SIP_S2_9 had mean 24-hour sleep times below 250 min, and males of SIP_S2_1, SIP_S2_2, and SIP_S2_8 had mean 24-hour sleep under 300 min (Figure 2H). These short sleep times rival flies with single-gene mutations in *Shaker* (247 ± 22 min for females and 297 ± 34 for males) (Cirelli *et al.* 2005), *insomniac* (317 min for males) (Stavropoulos and Young 2011), and *sleepless* (Koh *et al.* 2008). Remarkably, all S2-derived short-sleeping lines had night sleep that was significantly lower than the shortest-sleeping line of the DGRP, DGRP_38, and all but two had shorter 24-hour sleep (Figure S4) (Harbison *et al.* 2013). The S1-derived lines SIP_S1_1 and SIP_S1_2 had shorter 24-hour sleep as well (Figure S4). Although night sleep in all but one of the long-sleeper lines was the same as the longest-sleeping line of the DGRP (Figure S4), DGRP_335, 24-hour sleep in DGRP_335 was significantly longer than all of the SIP lines (Figure S4). This is likely due to the fact that the selection procedure targeted only night sleep (Harbison *et al.* 2017); while day and night sleep share some genetic architecture, day sleep is not completely correlated with night sleep (Harbison *et al.* 2009; Harbison *et al.* 2013). In addition, we found other sleep traits with significant differences between long- and short-sleep selection schemes, which included day sleep duration (*P*_Selection_ = 0.0057, four-way nested ANOVA), sleep latency (*P*_Selection_ = 0.0386, four-way nested ANOVA), and average night bout length (marginally significant *P*_Selection_ = 0.0553, four-way nested ANOVA). The differences in these sleep parameters between the long and short sleepers reflected correlated responses that we observed in the progenitor populations (Harbison *et al.* 2017). Stable extreme long and short sleeping phenotypes can therefore be constructed from naturally-occurring variants.

### Short sleeper lines of the SIP have more variable day-to-day sleep

We previously noted strong negative correlations between the variability in sleep among individual flies and both night and day sleep duration (Harbison *et al.* 2013; Harbison *et al.* 2017); specifically, we found that shorter sleep times were associated with increased variability in sleep duration among flies. We calculated the variability in sleep parameters among individual flies of the SIP as the coefficient of environmental variation, or *CV*_E_ (Table S5) (Mackay and Lyman 2005). None of the *CV*_E_ traits were significantly different by selection scheme, suggesting that long sleepers and short sleepers had the same overall inter-individual variability, though night sleep *CV*_E_, day sleep *CV*_E_, 24-hour sleep *CV*_E_, and day bout number *CV*_E_ were close to significance (Table S1 and S6). However, when we examined daily fluctuations in sleep using the standard deviation of each sleep trait (σ) to represent intra-individual differences (Knutson *et al.* 2007; Mezick *et al.* 2009; Buman *et al.* 2011; Angulo-Barroso *et al.* 2013; Dillon *et al.* 2014), we found that night and 24-hour sleep σ and night bout number σ were increased in lines derived from short-sleeping populations, and reduced in lines derived from the long-sleeping populations (*P* = 0.0398, 0.0428, and 0.0312, respectively, 3-way ANOVA) (Tables S1, S7, and S8). Short sleepers, therefore, have more daily fluctuations in sleep than long sleepers, and their sleep also tends to differ from individual to individual (Harbison *et al.* 2013; Wu *et al.* 2018). We speculate that the short sleepers may have greater sensitivity to small environmental fluctuations, and that this may result in more variable sleep.

### Genomic architecture of the SIP

We extracted DNA from female flies and sequenced a minimum of 10 million 251 bp paired-end reads per SIP line, producing 30-50X genome coverage on average (Figure S1). We counted polymorphic variants and small indels known to be segregating in the 10 DGRP lines used to create the SAIP (Mackay *et al.* 2012; Huang *et al.* 2014). In addition, we searched for potential *de novo* variants using LoFreq (Wilm *et al.* 2012). LoFreq detected 1,451,085 (BWA alignment) and 1,298,672 (Novoalign alignment) variants. Results were similar for each sample’s BWA and Novoalign alignment sets, with less than 3% difference among the variants called for the *X*, *2L*, *2R*, *3L*, *3R*, and *4* chromosome arms, while differences between the two alignments were 20% and 22.8% for the mitochondrial genome and the *Y* chromosome, respectively (Table S2). Most of these variants were known DGRP SNPs (80.9% BWA and 65% Novoalign) (Table S9). We used the distribution of the LoFreq quality scores for the known DGRP SNPs to find a quality score threshold (1000) for the remaining SNPs (Figure S2). We eliminated 247,228 BWA SNPs and 432,612 Novoalign SNPs having quality scores less than 1000 from the final set of variants. We found 2,810 putative novel variants using the BWA alignment and 1,197 with Novoalign that appear to have arisen in the 10 DGRP founders. Furthermore, we found 183 novel variants (BWA) and 114 variants (Novoalign) that were restricted to one artificial population only (*i.e.*, L1, L2, S1, or S2). The numbers of novel variants were reasonable given a recent study of the accumulation of mutations over 60 generations in a single DGRP line (Huang *et al.* 2016); in that study, the spontaneous mutation rate was estimated as 6.96 × 10^−9^ for the *X* chromosome and 6.25 × 10^−9^ for the autosomes, giving 1,456 *de novo* mutations. The remaining SNPs mapped to the *4*, *M*, or *Y* chromosomes or regions not well defined in the *D. melanogaster* version 5.0 sequence used to call variants in the DGRP. We therefore consider it likely that these variants are part of the 10 DGRP founder genomes. Thus, nearly all the variants that we found map to the DGRP founder lines.

**Figure 3:**
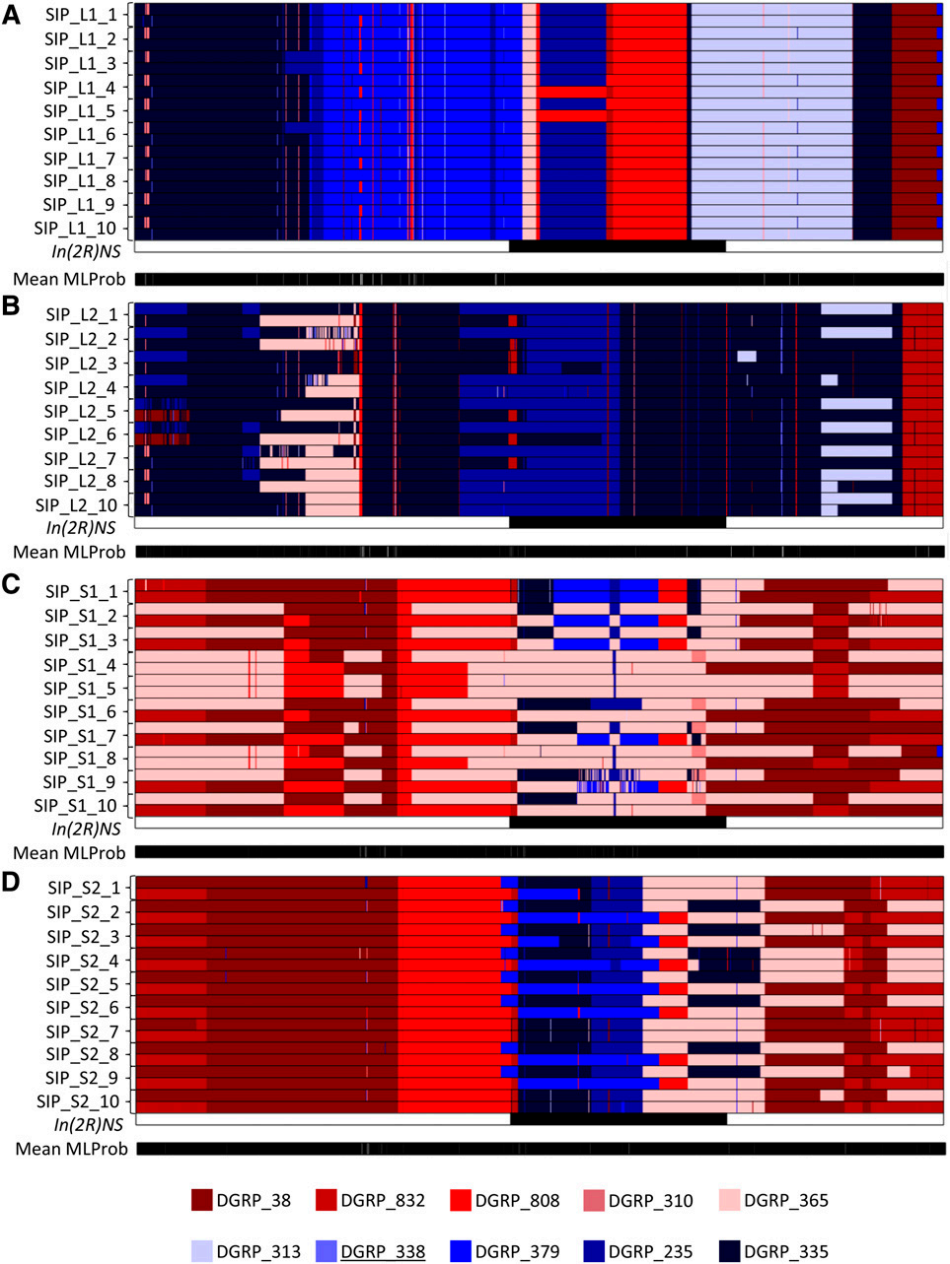
Plot of predicted DGRP founder haplotypes on chromosome *2R* from Hidden Markov Model. In each plot, the predicted founder haplotypes are plotted along the length of the chromosome. Short-sleeping founder genotypes are coded in shades of red per the legend at the bottom of the figure; long-sleeping founder lines are coded in shades of blue. The location of chromosomal inversions is indicated by black bars for inversions that were present in the 10 DGRP founder lines. Underlined DGRP lines listed in the legend are heterozygous for the indicated inversion. The average posterior probability is given as a bar underneath the chromosome schematic graded from a probability of 1.0 (black) to 0.0 (white). (A), Lines derived from L1; (B), Lines derived from L2; (C), Lines derived from S1; (D), Lines derived from S2.

We used a Hidden Markov Model (King *et al.* 2012) to infer the distribution of the 10 founder DGRP lines along the chromosomes of each SIP line. The model performed well on our data, predicting founder states with posterior probabilities of at least 0.95 on 93.8% of our model’s sites. The fact that founder states were confidently predicted by the HMM suggested that contamination by other genotypes at any stage of the experiment (initial crosses, selection, post-selection maintenance, and inbreeding) was very unlikely. We plotted the inferred genotypes along each chromosome arm of the SIP (chromosome *2R*, Figure 3; remaining major chromosome arms, File S1). Figure 3 shows how the founder lines combined to make chromosome *2R* in the lines of the SIP. As expected, the greater contribution of the long-sleeping DGRP lines (shaded in hues of blue) can be seen in the L1 and L2 SIP lines (Figure 3A and 3B), while the shorter-sleeping DGRP lines (shaded in reds) contributed more to the S1 and S2 SIP lines (Figure 3C and 3D). The figure also shows the location of an inversion, *In(2R)NS*, which was heterozygous in one of the founder lines, DGRP_338 (Huang *et al.* 2014). This inversion does not appear to be present in any of the SIP lines. In addition, the average posterior probabilities are plotted along chromosome length. Brief switches of founder genotype tended to be associated with lower posterior probabilities. Thus, with the HMM model, the overall contribution of each of the original 10 founder DGRP lines can be observed.

The contribution of these founder lines enabled us to compare the homozygosity of the SIP lines to that of the original DGRP founders. While the predicted founder haplotype for a given SNP was often heterozygous (*i.e.*, DGRP_38 and DGRP_832), the SNP alleles themselves were often homozygous. When we compared the actual allelic proportions of each variant of the SIP to the predicted founder alleles to assess homozygosity, the SIP lines were between 1.64% less to 2.14% more homozygous than the genotypes predicted by the HMM model (Table S10).

Here we have developed a panel of 39 inbred long- and short- sleeping lines, a resource that will be useful for developing phenotypic correlates, perturbing genomic variants, and assessing changes in gene expression and protein abundance. These lines are available through the Bloomington Drosophila Stock Center, and the sequences are available through the NCBI Sequence Read Archive.
